# Becoming a medical educator: motivation, socialisation and navigation

**DOI:** 10.1186/1472-6920-14-110

**Published:** 2014-05-31

**Authors:** Emma Bartle, Jill Thistlethwaite

**Affiliations:** 1 Centre for Medical Education Research and Scholarship, School of Medicine, The University of Queensland, 288 Herston Road, Herston, QLD 4006, Australia

## Abstract

**Background:**

Despite an increasing concern about a future shortage of medical educators, little published research exists on career choices in medical education nor the impact of specific training posts in medical education (e.g. academic registrar/resident positions). Medical educators at all levels, from both medical and non-medical backgrounds, are crucial for the training of medical students, junior doctors and in continuing professional development. We explored the motivations and experiences of junior doctors considering an education career and undertaking a medical education registrar (MER) post.

**Methods:**

Data were collected through semi-structured interviews with junior doctors and clinicians across Queensland Health. Framework analysis was used to identify themes in the data, based on our defined research questions and the medical education workforce issues prompting the study. We applied socio-cognitive career theory to guide our analysis and to explore the experience of junior doctors in medical education registrar posts as they enter, navigate and fulfil the role.

**Results:**

We identified six key themes in the data: motivation for career choice and wanting to provide better education; personal goals, expectations and the need for self-direction; the influence of role models; defining one’s identity; support networks and the need for research as a potential barrier to pursuing a career in/with education. We also identified the similarities and differences between the MERs’ experiences to develop a composite of an MER’s journey through career choice, experience in role and outcomes.

**Conclusions:**

There is growing interest from junior doctors in pursuing education pathways in a clinical environment. They want to enhance clinical teaching in the hospitals and become specialists with an interest in education, and have no particular interest in research or academia. This has implications for the recruitment and training of the next generation of clinical educators.

## Background

There is increasing concern about a medical education workforce shortage [1]. Currently, the education and training of medical students and junior doctors is delivered by a combination of university-employed academics (either medically or non-medically trained), clinicians with academic titles and both health service and university contracts, and health practitioners, mainly doctors, employed solely by health service providers. The principal role of this last group is service provision, often in high workload clinical environments.

Doctors have always had a commitment to teaching; indeed this is a well recognised component of medical professionalism. This responsibility is further professionalised in both the foundation curriculum in the UK (‘demonstrates the knowledge, skills, attitudes and behaviours to undertake a teaching role’ [2]) and the Australian curriculum framework for junior doctors (‘plans, develops and conducts teaching sessions for peers and juniors; uses varied approaches to teaching small and large groups; incorporates teaching into clinical work; evaluates and responds to feedback on own teaching’ [3]), which include teaching as a core competence for all junior doctors, regardless of their career choices. Some of these doctors will become motivated to spend a greater proportion of their time in education, and will seek out opportunities to do this, including taking up academic positions.

Academic medicine is founded on the three pillars of clinical service, research and teaching, and the interrelationships between them [4]. However, whilst an academic department of a university is the natural home of the clinician researcher, the advantages of belonging to a university are not so apparent for the clinician educator; professional socialisation into education therefore involves a diverse range of pathways. The deterrents to pursuing an academic career both in relation to research and teaching have been summarised, for example by Walport in the UK, [5] as a lack of clear entry routes, structured career pathways, flexibility in terms of the geography of available places, balance of work between service and academia (and life), and the availability of properly structured and funded posts on completion of training. Additionally, as with academic careers in other sectors, the success of clinical educators is measured in terms of research productivity and clinical service rather than teaching. As Harmon notes, it is research that is traditionally perceived to be the unique contribution of university-based academia to the medical profession and wider community [6]. We also note a lack of consensus as to the definition of a ‘medical educator’.

A study of clinician educators at the University of Sydney concluded that the current focus on socialising clinicians into academic medicine in terms of research would need to change substantially to facilitate the rise of the clinician educator [7]. The study indicated that the discipline structure of academic medicine and the research-focused culture of academic and institutional expectations could engender feelings of inauthenticity and marginalisation for those clinicians who favour teaching over research.

Of interest on the continuum of clinician-educator roles are the junior doctors who have an interest in teaching and who wish to develop a career in education alongside their clinical roles. They may not want to pursue an academic pathway through a university appointment but may wish to be recognised as having enhanced skills in education in the clinical setting. There is little in the literature about their experiences, their socialisation and navigation as educators in service environments or about any possible influence of their curriculum in motivating their interest in teaching. This paper explores the experience of junior doctors in medical education registrar posts as they enter, navigate and fulfil the role.

### The context of this study

The setting for this study was Queensland, Australia. In the last decade the Australian government has significantly increased the number of medical students through the expansion of existing medical schools and funding of new schools. This has led to a marked rise in the number of medical graduates [8] and the necessity to enhance teaching and supervisory capacity for both students and junior doctors through the development of clinical educators [9]. In 2007, Queensland Health (the funder and supplier of the state’s health service, with a network of 17 hospital and health service districts across Queensland) identified its own need to build capacity in the area of clinical education and training [10] and in 2008 developed the Medical Education Registrar (MER) scholarship program for this purpose (Table 1). Medical Workforce Advice and Coordination (MWAC) provided funding for four fulltime hospital-based MER positions per year across Queensland from 2008–2012. Several health service districts funded their own positions in addition to this: two in 2009, two in 2010, one in 2011 and seven in 2012. The last of the MWAC-funded positions finished at the end of 2012. There were nine applications across the state for the four MWAC-funded positions in 2012.

**Table 1:** **The objectives of the MER position **[11]

**The key activities of the registrar are to:**	
1. Develop an understanding of the role and functions of the Director of Clinical Training.	4. Disseminate knowledge and skills in the area of medical education to consultants, registrars and junior doctors.
2. Develop a more thorough understanding of educational theory and practice as it pertains to the clinical education and training of doctors.	5. Contribute to the field of medical education through research activities and other projects.
3. Assist in the development and planning of educational activities for junior doctors.	6. Undertake at least one clinical shift per week.

The MER positions were designed as a 12-month period of developmental experience for junior doctors, within the context of the registrar (resident) career continuum. The positions aimed to allow registrars to undertake a training program of activities to develop clinical education knowledge and skills, including supervisory skills, while continuing to do one or two sessions of clinical work in their specialty per week. There was also some expectation that they would undertake an education project and thus contribute to medical education scholarship, though the nature and type of this was not specified. Overall the program aimed to develop a community of educators to enhance the learning and teaching culture within clinical environments.

The role of the MER was considered to have great potential to build education capacity in the clinical context and a state wide selection process was used. MERs were selected based on their planned project and other activities, and the capacity of their supervisors to support and facilitate an effective experience in medical education. Queensland Medical Education and Training (QMET) provided additional educational support during the scholarship term through four bimonthly Medical Education Registrar and Supervisor (MERSS) Forums. (At the time QMET was the division of Queensland Health responsible for overseeing strategy and coordination of medical education and training activities for clinicians within Queensland).

The last MWAC-funded fulltime MER positions finished at the end of 2012. From 2013 hospitals/districts may use some of their own training money to support education registrars, however this is optional. The increasing number of applicants each year for the MWAC-funded MER posts indicates a growing interest from junior doctors in this career pathway.

Given the current concern about a shortage of medical and clinical educators [1], this study is timely and aims to explore the motivations and experiences of junior doctors wishing to pursue an education career pathway firstly through a non-academic post. We were interested in the outcomes of these posts in terms of plans to continue as an educator, and whether such plans would involve an academic career and why/why not. Our specific research questions are:

•What influences junior doctors to become educators?

•What do the career pathways of junior doctors as educators look like and what factors facilitate or hinder this journey?

•What experiences do junior clinician educators have during their professional socialisation as educators?

•How do these junior doctors describe themselves and their profession?

•What are the implications for junior doctor training of the findings of this project?

### Theoretical framework

As discussed further below we adopted socio-cognitive career theory (SCCT) as a framework for the data analysis as we read through the transcripts. SCCT has been used to explore issues associated with choice of career pathways in a number of disciplines [12], including academic medicine [7]. Derived primarily from Bandura’s general social cognitive theory [13], SCCT provides a useful conceptual framework for understanding the interplay between an individual’s career interests, choice and performance, as well as understanding how personal factors, such as outcome expectations, self-efficacy beliefs and personal goals, can interrelate with contextual and experiential supports or barriers [12]. SCCT assumes that individuals require both component skills and a strong sense of self-efficacy to achieve competent performance [14].

Self-efficacy is used to describe people’s self-judgements of their capability to perform a role and succeed in specific situations, and can be influenced by other people, observational learning, behaviour and contextual factors [13,15]. In particular, self-efficacy beliefs are thought to impact on an individual’s activity choice, effort and persistence, thought patterns, and emotional reactions [12]. Self-efficacy has been found to be predictive of academic and career-related choice and performance [16-18].

Outcome expectations refers to an individual’s estimate that a specific behaviour within a given context will lead to certain outcomes [12,13]. Personal goals describe the personal, professional and lifestyle goals set by individuals to organise their behaviour and guide their actions. A complementary construct is the notion of career barriers, personal and contextual factors which result in dissonance among career aspirations, progress and achievement. Overall, career barriers engender negative outcome expectations in those contemplating a particular career pathway [19].

Relatively few SCCT studies have employed longitudinal designs within the context of academic careers. A study by Bakken et al. analysed the career development pathways for clinician researchers, using SCCT as a framework [20]. They found that the development of a clinical research pathway has many potential challenges, including low self-efficacy beliefs, over commitment, negative outcome expectations, ill-defined personal goals, and the conflicting demands and expectations of the multiple environments an individual may inhabit.

A more recent study by O’Sullivan et al. from the University of California School of Medicine reported similar findings, using SCCT to identify interaction with role models and mentors, visibility of career pathways, career support for junior staff, early exposure to research and the interplay between personal and social factors as key elements related to participants’ interest in becoming academic doctors [21]. Both papers employed SCCT to analyse the development of research rather than teaching pathways. Dornan has suggested that Bakken and colleagues’ formulation could be extended to ‘the whole breadth of a scholarly career that included education and tertiary practice’ [22].

## Methods

### Data collection

We conducted semi-structured interviews with 12 participants (out of a potential pool of 32 current and past MERs). Participants were recruited to the study via email invitation. They included 10 junior doctors undertaking a medical education registrar position during 2012 at one of the Queensland hospitals and two doctors who had completed the posts. Three of the participants completed their MER post at regional hospitals and the other nine at major metropolitan hospitals. Interviews with participants were conducted at either one or both of two time-points: firstly, during the early weeks of eight MERs in the programme in 2012 and, secondly, as follow-up interviews with five of these MERs conducted upon completion of their post. Not all of the MERs were available for follow-up interviews. The sample also included two clinicians who had completed a medical education registrar role between 2009 and 2010 and who were interviewed retrospectively to gain a richer insight into any longer term impact of the posts, making a total of 15 interviews. The male: female gender ratio among participants was 5:7. Given the small sample size, participant characteristics are not fully identified in this study to protect anonymity. Neither author had any prior relationship with the participants.

We developed an interview question guide, ensuring that a level of consistency in the broad topics covered was maintained across the three interviewers (the two authors and a research assistant). The guide was informed by the literature and for the later interviews by SCCT. The interviews broadly explored participants’ motivations and experiences of entering and pursuing an academic career generally and, more specifically, a teaching career pathway, their motivation, the main challenges and barriers encountered in teaching, the support networks available and the further support required, the balance between teaching and clinical, perceptions of their role by others, self-identity within a clinical environment and future career pathways. Open questions related to the topics above were used to draw out issues of significance to respondents. Ethical approval was obtained for the study through the Queensland Health Human Research Ethics Committee (Reference no: HREC/11/QHC/51).

### Data analysis

The interviews were transcribed verbatim and firstly analysed by the two authors and the research assistant using framework analysis [23], a deductive approach based on our defined research questions and the medical education workforce issues prompting the study. Initially, two transcripts were analysed by each of the three members of the research team to begin to identify a thematic framework. Each member then independently applied the framework to all the interview data through a coding process. Next, we compared and contrasted coding, and discussed the value of using SCCT as the theoretical framework given the themes that were emerging from the data. Subsequently SCCT informed data collection for the follow-up interviews. The SCCT framework was applied to a portion of the dataset by the two authors to establish validity and check for new themes. The final framework was applied to the whole dataset by one author. The association between themes, comparison with SCCT and interpretation of the findings were discussed by authors both to finalise the text presented. Our study adheres to the RATS guidelines for reporting qualitative studies. The authors each used a different piece of software for qualitative data analysis and management (NVivo and Atlas^TI^). This was based on each author’s personal preference and we do not believe that it has theoretical implications in our analysis.

## Results

The MERs are identified by number: 1–12; 1a through to 8a were interviewed in the early stages of the posts; 1b and 7b through to 12b were interviewed after they had completed their terms. Themes identified in the data are listed in Table 2.

**Table 2:** Themes arising from the data

• Motivation for career choice: wanting to provide better education	• Defining one’s identity
• Personal goals, expectations and the need for self-direction	• Supports: supervisors and mentors
• The influence of role models	• Potential barriers to pursuing a career in/with education: the need for research

### Motivation for career choice: wanting to provide better education

The registrars expressed a general interest in teaching often fuelled by a strong sense of the impact of either their own excellent or (more frequently) poor experiences of education or from their observation of clinical teachers interacting with others. Their negative experiences in particular motivated their interest in being able to teach better than their own teachers. Their sense of self-efficacy prompted the belief that they could emulate their good teachers while performing better than those they recognised as having few pedagogical skills.

‘But I think anybody who has junior doctors underneath them should have the responsibility to get some experience or some sort of ability to teach.’ (8b)

However following their experience of actually being teachers during the MER term, while identifying that teaching is a fundamental role of the senior clinician, they highlighted that it is a skill that needs nurturing rather than something that would come naturally to a doctor:

Teaching is not one of those things you can just pick up and do. I think a lot of people quite wrongly assume that just because you're a doctor or just because you're a surgeon or just because you're a physician that you can teach your subject matter and you can't. Sorry, I think you know, sometimes you need to have a different set of skills.’ (8b)

### Personal goals, expectations and the need for self-direction

While the MERs had career goals and the aim of developing as better educators as motivating factors to undertake the MER position, many had not considered what they otherwise hoped to achieve during the post itself. This lack of clarity was compounded by the fact that some MERs had not received a formal role statement or job description or could not recall having done so. It appears the QMET-defined objectives for the position were not provided to all of the MERs at the start of their post. Moreover it was during the interview for this study that some of them first began to think about how they would evaluate the outcome of the post for themselves and their institution. This lack of goal setting, or ill-defined goals, was partly related to the lack of orientation at the start of the post and partly due to the need for self-direction in determining just what was possible and permissible during the position itself:

‘…because it's not clearly defined in terms of you will do this, this, this - it's very broadly allocated to that - it's allowed me to do all of these different things that I've got a personal interest with.’ (7a)

‘It wasn't until one of the previous people, who have done the same post, said that once she embraced the fact that she was the master of her own destiny so to speak for the year - once she accepted that, then she just got on with it and did it. I think talking to her about that and hearing that, really helped. So I've just started to be more - well, I'm just going to do this and I'm going to do this. Whereas before I was waiting, as though maybe someone needed to tell me exactly what I needed to do.’ (2a)

However in retrospect this self-direction was seen as an advantage:

‘I thought one of the challenges was that there wasn't very much direction. In the end, I think that was actually really a huge positive for me, because it allowed me to do what I wanted.’ (9b)

The lack of understanding of the role by their peers and more senior clinicians could be frustrating:

‘Other times where people have asked me to do things that I've looked at and thought, I don't quite see how that marries with medical education.’ (7a)

‘Some hospitals also interpreted medical education as medical admin and so we found that some registrars were doing administration which is not part of it obviously.’ (12b)

The contrast between the heavy workload of a clinical rotation and the self-direction required as a MER made for a difficult period of transition, either because of the luxury of time or the balancing act required for the number of roles within the position:

‘It was very difficult initially coming from such a time pressured role in all of my previous years where you don't get lunch, you know, you're a clinician, you're running around or you're doing your jobs and that's the way it is. Then to come to somewhere where you have an office and a computer of your own and you have time to think and contemplate and plan and research, that was very, very difficult. I found that really difficult for three months.’ (4a)

‘I am struggling to fit in all things. I have to juggle between things.’ (6a)

### The influence of role models

Role models took many guises: the people who had inspired some MERs to take up their posts, clinicians observed during training who were noted as excellent, inspiring and ‘natural’ teachers, and supervisors who were also seen as mentors and people to emulate in that respect:

‘The fact that they don't even know they're teaching half the time, it comes so naturally to them and they're not teaching esoteric stuff. That's my point of view is that these people that I view as mentors or role models have taught me the important stuff.’ (4a)

‘They're more role models as well - just great teaching styles; relaxed, not over pushy. I think that's probably the things that I've found that I've liked in most of the educators is a relaxed style where they're approachable and contactable, and just have interest in it…a whole combination of fairly simple things.’ (5a)

Having identified their role models, novices need to reflect on those role models’ attributes and behaviour in order to improve:

‘I just role modeled off other senior clinicians…that I found as good teachers and tried to work out what’s made them good teachers as opposed to other clinicians I didn’t find to be good teachers. That’s kind of made me strive to think about how my own ways of teaching and how I could make myself better.’ (3a).

### Defining one’s identity

The practice of medicine is a discipline with many potential pathways and professional identities. However the pathways and nomenclature are not always clear. The MERs emphasised the importance of continuing with their clinical identity in terms of credibility as an educator and their commitment to patient care:

“Probably from my own experience, if somebody was trying to teach me a skill or what have you and they weren’t working as a doctor at the time, I’d probably think, I’m not sure that you know what you’re talking about because you’re just sitting in an office all day. So seeing them as well in the clinical context is important to reinforce that you’re still working and what you’re saying is probably useful.’ (3a)

“I can't see myself as a medical educator, as an alternative to a clinician. I miss clinical work significantly and that's been a very significant downside of this role for me.’ (4a)

Some respondents’ role saw themselves as having multiple identities whereas others had much firmer ideas of their position in the medical community. Those MERs who had finished their terms tended to have more certainty in describing themselves:

‘It depends on the context of what I'm doing.’ (11b)

‘Definitely my professional identity is I'm a surgeon. I'm a surgeon with an interest in educating.’ (8b)

‘I'm a doctor but my role as a doctor is to teach.’ (12b)

Research as a component of the role or their identity was much less important:

‘I don't think, necessarily, to be a good clinician you need to be able to be a good researcher because I'm certainly not, and I'd hope that I'm a decent clinician. I do think the core things of teaching, because of the nature of our practice and the way that people are trained and the amount of people that come through, I think that's an essential part of being a good doctor and continuing the medical professional development; I think not so much the research, but certainly teaching clinical skills.’ (5a)

### Supports: supervisors and mentors

In terms of support and professional guidance the supervisor was the most important figure for the MERS, though this was somewhat complicated by having two supervisors during the term: the one responsible for the medical education part of the role (and their experience varied) and the other the discipline supervisor:

‘Well I've got my education supervisor and my surgical supervisor. They're basically the people I directly have anything to do with.’ (8b)

The attributes of a good supervisor were clearly defined, with a high degree of importance attached to feedback but also an acknowledgment of the autonomy required by an MER to get on with the job:

‘I do ask for feedback but it's a bit early to get proper feedback in terms of how I've done. I keep asking about how I'm doing and to tell me when I’m going wrong or what.’ (6a)

‘If you've got a good supervisor and you've got some imagination and you're passionate about education, I prefer it being a more open type of structure. If you're somebody that needs a lot of direction, I'm not sure that the medical education registrar position would be for you unless you had a very strong supervisor.’ (7a)

Not having a supervisor with sufficient knowledge of education was difficult and this was particularly an issue in relation to the expectation of doing the project:

‘Probably part of the reason that it was so frustrating…was the mentorship. I had people that I was talking to, who were very nice, but they didn't really have time or the knowledge of what I was actually doing. I don't think they had - it sounds terrible, but in some instances - not all, but some of the people that I was talking to had no more medical education experience then I did.’ (9b)

‘The biggest recurrent things are the registrars usually being too ambitious and potentially also an issue with supervision in either not being specific enough to really guide the learner or - and perhaps not recognising how ambitious a project may be but also the ability to have closer interaction with those supervisors.’ (12b)

### Potential barriers to pursuing education: the need for research

There was not much enthusiasm for pursuing medical education research. Research capability and delivery were seen as fundamental requirements for an academic career, and therefore the lack of interest in research was a barrier to academia but not necessarily to the clinician educator role. Moreover the necessity to do a higher degree to enter and progress in academia was particularly off-putting:

‘My views on research are that you shouldn't force people to do it. I think forcing research encourages bad research. I think when you find something that you are interested in and you see a need for some further work on it; I think that's the recipe for good research.’ (8b)

‘If I want to do particularly much more education, particularly in the university areas, I'm probably going to have to do a PhD…I accept that research is part of a higher qualification and I accept that research is a useful adjunct to education, but it's not my area of passion. It's the teaching.’ (9a)

‘Once you’ve done your time and your hard work to get your fellowship [specialty qualification] and you’re working as a consultant and finally earning some money, to go back to – especially without a PhD and stuff – to a university background would be a big step backwards.’ (10b)

Even though there had been courses on reading and interpreting research papers during medical school, there had been little exposure or opportunity to get involved in research prior to the MER role:

‘I’ve had the opportunity this year to do a bit of both, so education research and research in the [X] department. Both exposures have been very challenging. It’s been quite an eye opener as to how difficult it is and how poorly a lot of research is done.’ (3a)

‘Research, obviously, can be useful and has huge amounts of use, but I haven't had enough exposure to it.’ (5a)

There was also lack of capability or confidence to undertake evaluation:

‘I am not very understanding yet of how you assess an educational program in a clinical sphere… I struggle with understanding how you can evaluate things.’ (4a)

‘So I actually did a proper evaluation; went through the ethics application and all that sort of stuff and looking to how to evaluate the program. Unfortunately what I realised is to evaluate an educational program is difficult irrespective of who you talk to. Most of the evaluations are done at the lowest level, and that is consumer satisfaction or consumer confidence.’ (8b)

## Discussion

**Figure 1:**
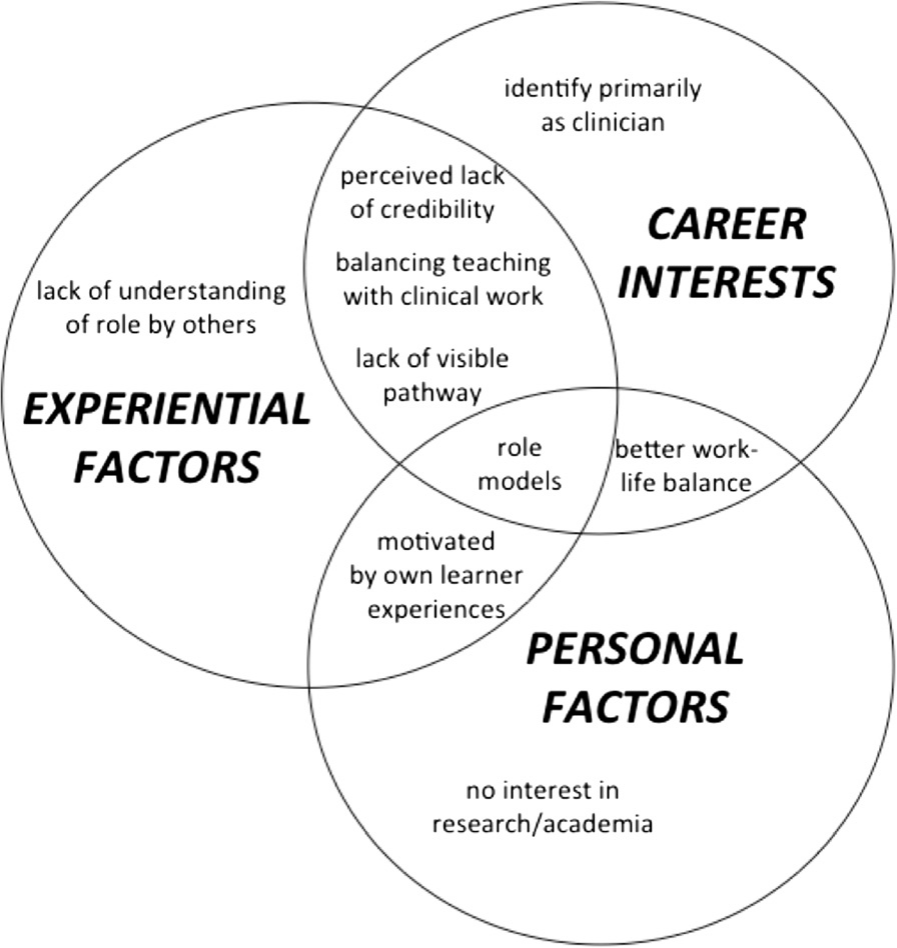
Alignment of themes to the SCCT framework.

Our findings illustrate the lack of career identity in relation to education. Interview data were analysed using socio-cognitive career theory [12,13] to explore the interplay between personal, contextual and experiential factors in relation to the experiences of junior doctors pursuing an education career pathway through a non-academic post (Figure 1). Motivation for wanting to provide better education, personal goals and expectations, the influence of role models, identity formation, and supports and barriers were the key themes to arise from the data.

The typical narrative of participants was a specialist registrar who fell into the MER post almost by accident and did not know much about what it entailed to start with. Regardless of how the participants heard about the position, and whether or not it was something they had aspired to for awhile, there was great similarity in their underlying motivations for wanting to pursue an education career pathway. Role models were important in influencing junior doctors to become educators. Participants did not have a specific interest in education when first entering medicine, but rather developed it during their undergraduate training as a result of their own experiences as a learner. Participants were intrinsically motivated to develop as educators and provide a better learning experience for the next generation of doctors than they had had. This resonates with the findings of Dahlstrom et al. [24], who identified a primary motivation for clinicians to engage in teaching was a desire to help educate the next generation of doctors [24]. Aligning with SCCT [22], the participants’ sense of self-efficacy to perform this was largely shaped by their own observations of other clinical teachers’ interactions with students and the influence of past role models.

Despite their initial beliefs that they could perform better than their own teachers, as participants navigated the role there was gradual recognition that teaching is a skill that needs to be nurtured and developed and does not necessarily come naturally. Recent studies on the developmental needs of junior doctors entering academic medicine have identified the provision of role models and creation of research opportunities as key requirements [21,22,25,26], yet there has been little discussion on the need to develop teaching skills. Our findings testify to the need to provide formal opportunities for junior doctors to learn these from senior medical educators, to facilitate their development as medical educators. This could require time away from clinical work, something that could negatively impact a MERs motivation to participate in this type of activity. It would also require recognition by colleagues of the credibility of an education career pathway; our findings illustrate there was no clear understanding of the MER roles by others in the system and they can be seen as a soft option. The failure of colleagues to recognise the MER positions as credible, negatively impacted on the junior doctors’ professional socialisation as educators.

The sense of identity emerged as a strong theme from the data. The MERs were not motivated by the chance to develop an academic career but wished to be seen as doctors with an interest and skill in education while working as specialists. These junior doctors primarily described themselves as clinicians; the identity of educator was seen as secondary to their main role. A consistent concern about the primacy of clinical work was expressed, and some respondents felt a strong pull back to full time clinical practice. When discussing identity, the role of an educator in a clinical environment, though often described as complementary, was almost always implicitly viewed as secondary to that of clinician. The lack of acknowledgement by peers of the validity of the role contributed to a dissonance between career aspirations and achievement. Misalignment between personal and contextual factors such as these have been reported to engender negative outcome expectations for those considering a specific career pathway [19,27].

The purpose of the MER position in different hospitals varied between sites based on hospitals’ needs, while also being capable of being tailored quite closely to the motivations of each MER. This different experience of structure and autonomy than in clinical roles was challenging for some participants as they navigated the role. However the lack of a formal position description was useful for those who were self-motivated and able to decide how they wanted to pursue their time. Certainly orientation would have been helpful and particularly a chance to talk to others in the role of those who have had the role previously.

The need for research was the biggest barrier to participants when considering whether to continue on a medical education career pathway upon completion of the post. There was not much enthusiasm for pursuing medical education research. Unlike the findings of O’Sullivan et al. whose respondents spoke of early exposure to research opportunities, our participants had little practical research experience other than an introduction to critical appraisal at medical school. This meant they had limited ideas of how to evaluate educational interventions or indeed gauge the success of their own projects. Teaching, hands-on and development, was the main objective and there was no particular interest in research or academia. They want to enhance clinical teaching in the hospitals and become specialists with an interest in education. This interest may also be of use for them to stand out from others applying for hospital posts. These findings support the recommendations of Kumar et al.[7], who in a recent study of clinician educators at the University of Sydney concluded that to facilitate the rise of the clinician educator, the current focus on socialising clinicians into medical education in terms of research will need to change substantially.

## Conclusions

There is growing interest from junior doctors in pursuing education pathways in a clinical environment. Continuing with their clinical identity is seen to be important in terms of credibility as an educator and a lack of interest in research means they have no desire to enter academia. There is a perceived lack of support for a blended clinician educator role in the hospital environment, with junior doctors feeling they ought to be either primarily a clinician or an academic. Medical educators at all levels are crucial for training and given the current concerns about a future shortage of clinical educators, it is crucial that a non-academic clinical educator is recognised as a legitimate career choice and opportunities to pursue this career pathway exist in the clinical environment, such as through the provision of defined training posts.

When providing training posts in medical education it is important to tailor to need and enhance the suitability of these posts in helping doctors develop educational skills. We have identified that medical education registrars need to be better supported in the clinical environment. An orientation at the start of the post would assist them to define personal goals for the post and understand expected outcomes. Providing a supervisor with sufficient knowledge of education would also better support MERs to evaluate their own projects and other educational interventions. There is a need to increase awareness of these roles in the health system, to ensure their credibility and enhance the learning and teaching culture within clinical environments.

### Limitations of the study

This is one of the first studies to explore the motivations and experiences of junior doctors entering a medical education career pathway within a clinical environment. Its key limitation is the fact that the small sample size was drawn from employees within one organisation, Queensland Health.

### Ethical approval

This study was approved by the Queensland Health Ethics Committee.

## Competing interests

The authors declare that they have no competing interests.

## Authors’ contributions

JT designed the study. Both authors participated in the recruitment into the study, data collection, data analysis and interpretation. Both authors contributed significant intellectual input to paper drafts and approved the final version of the paper.

## Pre-publication history

The pre-publication history for this paper can be accessed here:

http://www.biomedcentral.com/1472-6920/14/110/prepub
